# Thermo-Mechanical Behavior of Carbon Fiber Composites Processed at Elevated Temperatures

**DOI:** 10.3390/polym18030401

**Published:** 2026-02-03

**Authors:** Larisa-Anda Stroe, Daniel-Eugeniu Crunteanu, Mihail Botan, Adriana Stefan, George Catalin Cristea

**Affiliations:** 1 Faculty of Aerospace Engineering, National University of Science and Technology POLITEHNICA of Bucharest, Splaiul Independenţei 313, 060042 Bucharest, Romania; daniel.crunteanu@upb.ro; 2 INCAS—National Institute for Aerospace Research “Elie Carafoli”, 220 Iuliu Maniu Blvd, 061126 Bucharest, Romania; botan.mihail@incas.ro (M.B.);

**Keywords:** resin infusion, out-of-autoclave (OoA), mechanical properties, HDT, DSC, sustainable composite manufacturing

## Abstract

Out-of-autoclave (OoA) processing has emerged as a promising route for manufacturing high-performance polymer composites while reducing energy consumption and production complexity. The authors investigate the effect of curing temperature on the thermo-mechanical performances of carbon fiber-reinforced composites produced via resin infusion. Five laminates composed of six carbon fiber plies were arranged in a [90/0/45/−45/0/90] lay-up and infused with an epoxy resin cured at 25, 40, 50, 60, and 70 °C. The influence of the processed temperatures of the mechanical properties was evaluated through tensile and three-point bending tests, whereas thermal performance was analyzed using Heat Deflection Temperature (HDT) measurements and differential scanning calorimetry (DSC). The results demonstrate an improvement in stiffness, strength, and HDT with increasing the curing temperature, with the 40–50 °C range yielding the most balanced enhancement in mechanical and thermal responses. DSC analyses confirm that higher curing temperatures promote a more complete crosslinking reaction, consistent with the improved laminate performance. Overall, the findings highlight the critical role of controlled thermal curing in optimizing OoA polymer composite systems and support their suitability for energy-efficient applications.

## 1. Introduction

Carbon-fiber-reinforced polymers (CFRPs) play a central role in high-performance structures owing to their exceptional combination of specific strength and stiffness, superior fatigue behavior, and long-term durability in aggressive environments. These attributes enable substantial weight savings and enhanced structural efficiency, which are critical for applications in the aerospace, automotive, marine, and energy sectors [1,2,3,4]. Consequently, CFRPs are widely adopted wherever structural performance and lifecycle efficiency are of primary importance [5,6].

For several decades, autoclave processing has represented the reference manufacturing route for aerospace-grade composite materials. Elevated pressures and tightly controlled thermal cycles promote effective consolidation, low porosity levels, and reproducible mechanical properties [7,8], and still remain the benchmark for manufacturing high-performance thermoset composite structures due to the combined application of elevated temperature and external pressure, which results in excellent consolidation, low void contents, and reproducible mechanical properties suitable for aerospace structural components [9]. However, autoclave-based manufacturing is associated with significant industrial constraints, including very high capital and operating costs, considerable energy consumption, long processing cycles, and strict limitations on component size and geometric complexity. These drawbacks are increasingly incompatible with current demands for large-scale production and high manufacturing rates [9,10,11,12].

In response, out-of-autoclave manufacturing routes—particularly Resin Infusion—have rapidly emerged as credible alternatives for CFRP fabrication, especially for large panels and integrated structures [13]. In resin infusion under flexible tooling, dry fiber preforms are placed in a mold, sealed with a vacuum bag, and impregnated with liquid resin, with flow driven by pressure differentials [14]. This process is attractive due to its relatively simple tooling requirements, suitability for large and complex geometries, and significantly reduced production and energy costs compared with those of autoclave routes. When resin flow and curing parameters are properly controlled, laminate quality suitable for many engineering applications can be achieved [9,11]. As a result, resin-infused CFRPs are increasingly employed in secondary aerospace structures, automotive panels, and marine or energy components, where an optimal balance between performance, scalability, and cost is required [15,16].

Experimental studies indicate that resin infusion composites can achieve competitive tensile and flexural strengths when process conditions such as vacuum level, resin flow behavior, and curing temperature are carefully controlled, thereby narrowing the performance gap with autoclave-cured materials. For example, enhanced Vacuum-Assisted Resin Transfer Molding (VARTM) with controlled infusion and curing pressure increased tensile strength to approximately 760 MPa and maintained competitive flexural strength while reducing porosity below 1%, illustrating the potential for resin infusion to produce high-quality structural composites [17,18].

A comprehensive recent review by Shaik et al. (2021) [19] highlights the technological maturity of OoA composite manufacturing and emphasizes that resin infusion has evolved into a viable alternative for producing aerospace-grade thermoset composites. The authors report that optimized OoA routes, including resin infusion, can achieve structural quality and mechanical performance comparable to those of autoclave processing, while offering significant advantages in terms of manufacturing flexibility and cost efficiency [19].

Despite these advantages, the primary scientific and technological challenge associated with resin-infused composites remains defect control, particularly with respect to porosity and resin distribution uniformity. The literature frequently reports the occurrence of voids, resin-rich or resin-lean areas, and locally unimpregnated regions caused by entrapped air, reinforcement permeability variations, or non-uniform flow fronts. Such defects may lead to reductions in tensile and flexural strength and to the degradation of interlaminar performance. Divergent perspectives exist within the field: some studies demonstrate that optimized infusion strategies and suitable resin systems can substantially reduce porosity and yield mechanical properties similar to those of autoclave-processed composites for many structural applications, whereas other authors emphasize that such equivalence is highly sensitive to laminate thickness, flow design, and cure control, particularly for aerospace-relevant structures [13,20,21]. Establishing reliable relationships between processing parameters and final properties therefore remains essential for the safe structural design and broader industrial adoption of resin-infused CFRPs [22].

Qualification of these laminates requires rigorous mechanical and thermal characterization. Tensile testing enables the determination of the elastic modulus, ultimate strength, and failure strain, all of which are strongly influenced by fiber orientation and internal defects [23,24]. Flexural characterization using three-point bending is particularly relevant for quasi-isotropic laminates, as it simultaneously subjects the material to tensile and compressive stresses and highlights damage mechanisms such as matrix cracking, fiber micro-buckling, and delamination. From a thermal standpoint, the performance of epoxy-matrix CFRPs is limited by material softening near the glass transition temperature. Determination of the heat deflection temperature provides a practical threshold for stiffness loss under flexural loading and is widely used to define safe service windows. Differential scanning calorimetry complements these measurements by quantifying the glass transition temperature, changes in heat capacity, and cure-related thermal events, thereby enabling the assessment of crosslinking degree and polymer network stability [25,26].

Within this framework, the present study contributes to the ongoing transition from autoclave-dependent manufacturing toward optimized out-of-autoclave resin infusion technologies. The work focuses exclusively on CFRP laminates manufactured by resin infusion and investigates the influence of curing temperature on their mechanical and thermal behavior, providing a proof of concept for the TOPCOAT project based on heated-surface molds. Five quasi-isotropic carbon/epoxy samples with a [90/0/45/−45/0/90] stacking sequence were produced using identical infusion conditions, followed by two curing routes:

(i) Curing at ambient temperature;
(ii) Accelerated curing at 40, 50, 60 and 70 °C.

Test specimens were prepared by abrasive waterjet cutting to eliminate thermally affected zones and residual stresses that could bias experimental results [27]. The laminates were characterized through tensile and flexural mechanical testing, as well as thermal evaluation by heat deflection temperature measurements and calorimetric analysis.

The main objective of this work is to determine whether a moderate post-curing treatment at different temperatures up to 70 °C can sufficiently enhance epoxy crosslinking to yield improved mechanical performance and thermal stability compared with ambient-temperature curing. The results demonstrate that accelerated curing produces a more favorable balance of properties, with particularly notable improvements in flexural response and thermal stability.

Overall, these findings confirm that well-controlled resin infusion processes, combined with moderate post-curing enabled by heated molds, can deliver CFRP laminates suitable for demanding structural applications, while effectively avoiding the cost, scalability, and infrastructure penalties associated with autoclave-based manufacturing.

## 2. Materials and Methods

### 2.1. Composite Manufacturing by Resin Infusion

Carbon fiber-reinforced polymer composites offer high specific stiffness and strength; however, their performance is strongly governed by reinforcement architecture, impregnation quality, and the cure state of the polymer matrix. In OoA manufacturing routes, the absence of externally applied pressure increases sensitivity to resin flow behavior and cure kinetics, rendering porosity development and resin distribution key determinants of the final microstructure and mechanical response [28,29,30].

The reinforcement consisted of a twill carbon fiber fabric with an areal weight of 200 g/m^2^, supplied by Easy Composites Ltd. (Stoke-on-Trent, UK), selected for its stable in-plane mechanical behavior in thin structural laminates. The matrix system was a two-component epoxy resin based on L20 epoxy resin and H161 hardener, both supplied by R&G Faserverbundwerkstoffe GmbH (Waldenbuch, Germany). The resin and hardener were mixed at a mass ratio of 4:1, in accordance with the manufacturer’s recommendations, ensuring good compatibility with carbon fiber reinforcements and the development of stable mechanical properties after curing and post-curing.

Composite laminates were manufactured via resin infusion using a thermosetting epoxy matrix. Resin infusion is a liquid composite molding route in which low-viscosity epoxy resin is introduced into a dry fiber preform to achieve complete impregnation and laminate consolidation. The process supports effective fiber wetting and consistent laminate quality when key parameters such as resin viscosity evolution, permeability, and cure conditions are appropriately controlled, as discussed in recent epoxy-focused infusion studies [31,32,33].

**Figure 1:**
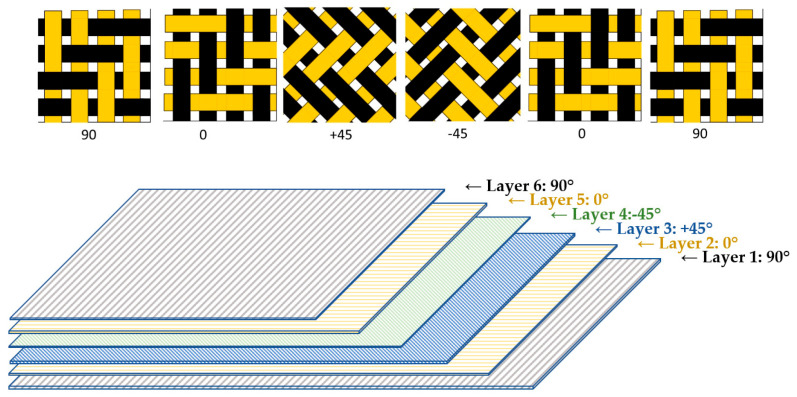
Stacking sequence of composite laminate: [90°/0°/+45°/−45°/0°/90°].

**Figure 2:**
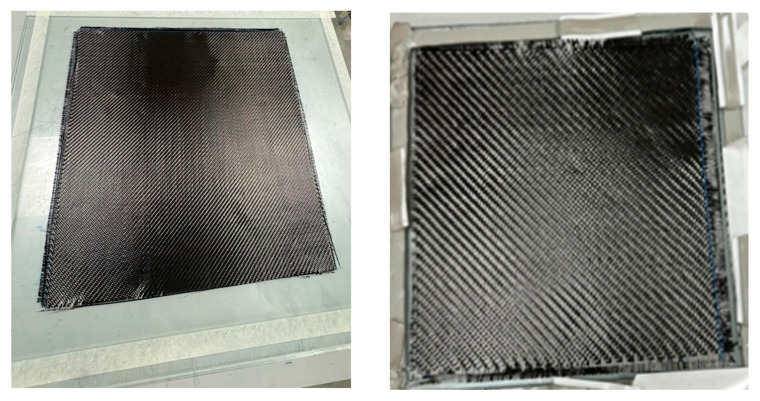
Dry composite laminate lay-ups illustrating the ply arrangement and surface morphology of representative specimens prior to resin infusion, manufactured using an identical fabrication procedure.

The plies were manually cut and stacked on a flat mold surface, with careful control of fiber orientation for each layer, as shown in Figure 1, using an identical balanced quasi-isotropic stacking sequence of [90°/0°/+45°/−45°/0°/90°]. The actual plies of representative laminate preform during the lay-up stage are illustrated in Figure 2, prior to resin infusion. While the two images show different laminate specimens, both are composed of six dry composite plies arranged according to the same stacking sequence as mentioned before.

This lay-up configuration was selected to ensure comparable in-plane stiffness and to minimize coupling effects under multiaxial loading conditions [34].

To evaluate the influence of curing temperature on thermo-mechanical performance, five composite panels were produced using identical materials, lay-up, and infusion parameters, differing only in their curing regime:

(i) Ambient-temperature curing for 24 h (25 ± 2 °C);
(ii) Curing for 12 h at 40 ± 2 °C on temperature-controlled heated surfaces;
(iii) Curing for 12 h at 50 ± 2 °C on temperature-controlled heated surfaces;
(iv) Curing for 12 h at 60 ± 2 °C on temperature-controlled heated surfaces;
(v) Curing for 12 h at 70 ± 2 °C on temperature-controlled heated surfaces.

**Figure 3:**
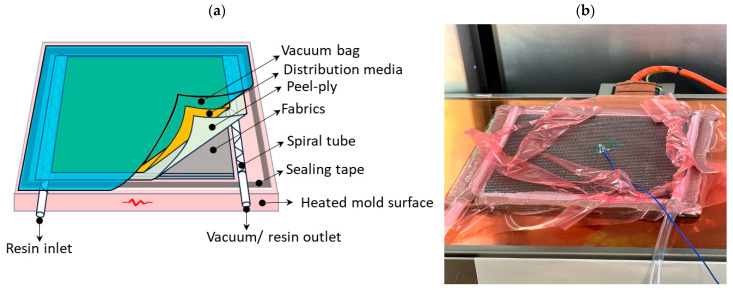
Schematic representation of the heat-assisted resin infusion process for fiber-reinforced composites (**a**), and representation of resin infusion during the thermal curing of the laminates (**b**).

The experimental framework was derived from the methodology developed within the TOPCOAT Research Project [35]. We reproduce the real-world application using heated molds for composite manufacturing and in this specific case using resin infusion process that is illustrated in Figure 3 [35]. Figure 3a shows a schematic representation of the dry carbon fiber fabric lay-up sealed under a vacuum bag during the heated resin infusion process, where the epoxy resin is drawn through the reinforcement by a pressure differential to ensure the complete impregnation of the fiber preform. Figure 3b illustrates the composite laminate after infusion, positioned on temperature-controlled heated surfaces, where thermal curing was carried out according to the prescribed curing regimes, T25, T40, T50, T60 and T70, where numbers mean the process temperature from the beginning of the infusion. With the T25 standard method from the manufacturer, the composite was held for 24 h and used as a reference for future comparison. Composites with the same carbon fiber stacking sequence were processed at 40, 50, 60, and 70 °C for 12 h. During the polymerization on top of each sample, a blanket was used for heat insulation and a thermocouple was used to record the difference in temperature to ensure a ±2 °C variation.

This processing route enables effective fiber wetting and laminate consolidation under out-of-autoclave conditions, while allowing precise control of the curing temperature [36].

Following the completion of the thermal curing cycle, all laminates were stored under ambient laboratory conditions for up to seven days prior to specimen preparation and testing. This conditioning stage allowed for additional room-temperature post-curing of the epoxy matrix, promoting the development of a stable and fully crosslinked network. By applying an identical conditioning duration to all panels, potential variability associated with incomplete post-curing was minimized.

The cured laminates exhibited a uniform final thickness of 1.25 mm ± 0.1 mm, reflecting precise process control and consistent material distribution across the laminate structure.

**Figure 4:**
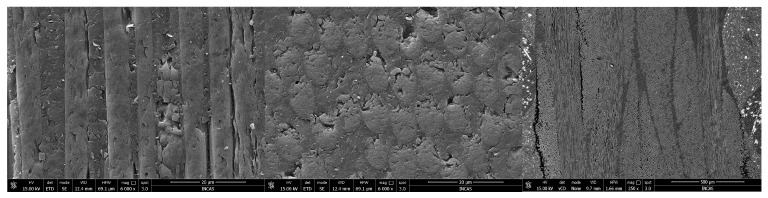
Cross-section SEM images of cured composites.

The composite laminate and carbon fiber after curing were observed by scanning electron microscopy (SEM) using a Quanta 250 microscope (FEI, Hillsboro, OR, USA), operated with the manufacturer’s dedicated xT Microscope Control Software for image acquisition and instrument control. From left to right, Figure 4 shows the longitudinal section of the carbon fiber (6000×), the transverse section (6000×), and the cross-section of cured composites (250×). SEM observations reveal good fiber–matrix adhesion, uniform fiber distribution, and the absence of significant voids or defects, indicating a high-quality lay-up and proper resin infiltration.

**Figure 5:**
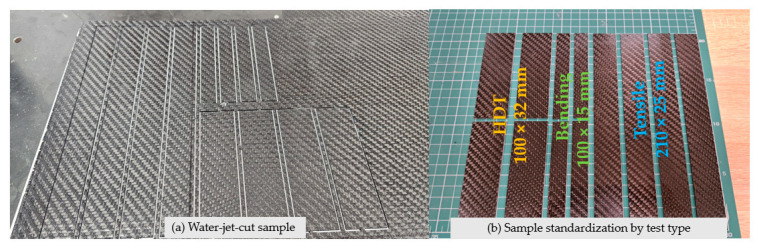
Standardized composite specimens prepared by abrasive waterjet cutting for tensile, flexural and HDT testing.

Subsequently, the cured composite panels were sectioned into test specimens using abrasive waterjet cutting with a WAZER Pro system manufactured by WAZER Inc. (Yonkers, NY, USA) [27], with toolpath generation and cutting parameters defined using the WAM 2.0 software. This cutting technique was selected to avoid thermal damage and to preserve the integrity of the fiber–matrix interface. As illustrated in Figure 5, the resulting specimens were geometrically standardized according to the requirements of the respective mechanical and thermo-mechanical characterization methods, including tensile and bending tests. In addition to the composite specimens, excess cured neat epoxy resin was collected at the end of the curing process and prepared for differential scanning calorimetry (DSC). Small bulk resin samples were extracted from the infusion spiral tubing cured with each respective temperature, trimmed to the exact mass.

### 2.2. Characterization Methods

Mechanical characterization was performed using a universal testing machine, Instron 5982 (Instron, Norwood, MA, USA), a high-precision static testing system suitable for a wide range of mechanical loading configurations. The machine was equipped with 10 kN and 100 kN load cells and controlled using Bluehill Universal^®^ V4 software for data acquisition and analysis. Axial tensile tests were carried out in accordance with ISO 527-4 [37], with continuous recording of force–displacement data. Stress–strain curves were derived using a non-contact video extensometer with a 200 mm field of view, allowing precise strain measurement while eliminating potential artifacts associated with mechanical contact between the extensometer and the specimens. This approach ensured the reliable evaluation of elastic modulus, tensile strength, and strain-at-failure values.

Flexural behavior was investigated using a three-point bending configuration, following standardized testing procedures. All flexural tests were performed at a constant crosshead speed of 2 mm/min. Load–deflection curves were analyzed to determine flexural stiffness, maximum load-bearing capacity, and the dominant failure mechanisms, including matrix cracking, interlaminar delamination, and brittle fracture behavior.

Thermo-mechanical performance was assessed through heat deflection temperature (HDT) testing using a Heat Deflection and Vicat Test System (HDT/Vicat) manufactured by Qualitest International Inc. (Plantation, FL, USA). HDT measurements were performed in accordance with ASTM D648 [38], under a constant flexural stress of 0.45 MPa. This stress level was achieved using a 3 N weight holder, corresponding to the standard loading condition specified by the test method. The specimens were subjected to controlled heating at a rate of 2 °C/min, and the temperature corresponding to the prescribed mid-span deflection was recorded as the heat deflection temperature.

For each curing condition and test category, a minimum of three specimens was tested to capture representative material variability and ensure an initial level of statistical reliability. This sample size reflects the exploratory scope of the present study, which focuses on identifying processing–structure–property relationships rather than full statistical qualification.

Thermal characterization of the epoxy system was performed using a DSC 300 Caliris differential scanning calorimeter (NETZSCH-Gerätebau GmbH, Selb, Germany), operated under a protective argon atmosphere and controlled and data analysis using NETZSCH PROTEUS^®^ V.9.5.3 software. Differential scanning calorimetry (DSC) measurements were conducted following the principles of ASTM D3418 [39] for the determination of glass transition temperature and cure-related thermal phenomena.

DSC analyses were carried out on cured L20 epoxy resin samples extracted from the composite laminates. Five curing conditions were investigated, corresponding to curing temperatures of 25, 40, 50, 60, and 70 °C. Sample masses ranged between 2.7 ± 1 mg, ensuring consistent thermal response. All measurements were performed over a temperature range of 30–200 °C using a heating–cooling protocol at a constant rate of 10 °C/min.

The heating segment of the DSC cycle was used to evaluate enthalpy relaxation phenomena associated with physical aging and residual molecular mobility within the epoxy network [40]. The characteristic temperature of this endothermic event was correlated with the heat deflection temperature obtained from thermo-mechanical testing. The cooling segment was employed to determine the glass transition temperature (Tg), defined as the midpoint of the heat capacity step. Determination of Tg from the cooling curve minimizes the influence of prior thermal history and provides a value representative of the effective crosslink density.

For each curing condition, three independent DSC measurements were performed. The reported DSC curves represent the averaged response (n = 3), ensuring improved reproducibility and reliability. This integrated mechanical and thermal characterization framework enables direct correlation between cure development, thermal stability, and the resulting thermo-mechanical performance of the composite laminates.

## 3. Results and Discussion

### 3.1. Tensile Behavior

**Figure 6:**
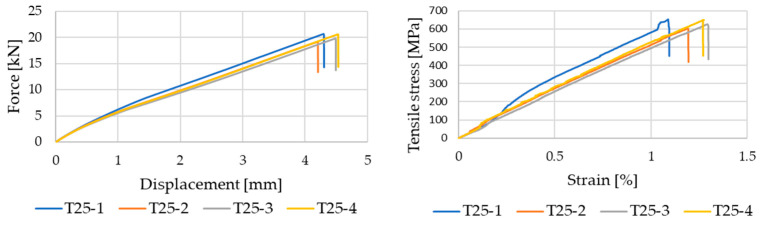
Tensile curves of crosslinked composite specimens at room temperature (25 °C).

The tensile tests performed on the out-of-autoclave CFRP laminates reveal a clear influence of the curing temperature on the mechanical response, as reflected by the force–displacement and stress–strain curves obtained for each curing condition. The tensile response obtained from the four tested specimens for samples cured at 25 °C is presented in Figure 6. This curing temperature was selected as the reference regime for the present study, and therefore all four individual tensile curves are intentionally reported to provide a detailed representation of the inherent repeatability and variability of the laminated composite sample under baseline conditions. For every curing regime, four individual tensile specimens were tested, as indicated by the multiple curves associated with each laminate. All specimens exhibit an initial linear elastic response followed by an abrupt failure, which is characteristic of fiber-dominated CFRP systems. The limited scatter observed among the individual curves can be attributed to local variations in laminate thickness, fiber distribution, and matrix continuity.

To ensure a robust and consistent comparison between the different curing regimes, the tensile properties discussed in this study are derived from the average values obtained from the four specimens tested for each crosslinked laminate. These mean values form the basis for the quantitative analysis of tensile strength, elastic modulus, and strain at failure as a function of curing temperature.

All laminates exhibit an initial linear elastic response followed by a sudden failure, typical of fiber-dominated CFRP systems. However, clear differences in stiffness, ultimate tensile strength, and strain at failure are observed as a function of curing regime.

**Figure 7:**
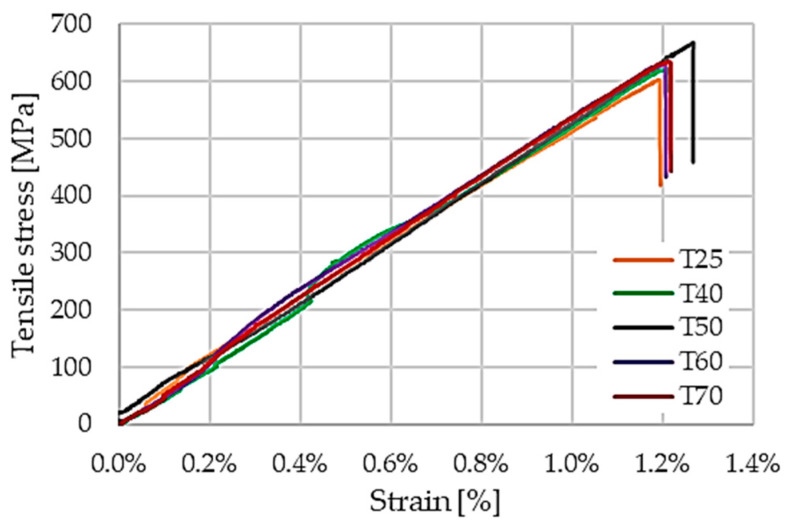
Tensile stress–strain curves of CFRP laminates cured at different temperatures.

Laminates cured at ambient temperature (25 °C) display a baseline tensile strength of approximately 630 MPa, combined with a moderate elastic modulus and tensile strain at failure exceeding 1.2%. Increasing the curing temperature to 40 °C and 50 °C leads to a noticeable improvement in tensile performance, with the highest average tensile strengths recorded in this temperature range. These trends are quantitatively summarized in Table 1 and comparatively illustrated in Figure 7, which reports the evolution of tensile strength, elastic modulus, and tensile strain at failure as a function of curing temperature.

**Figure 8:**
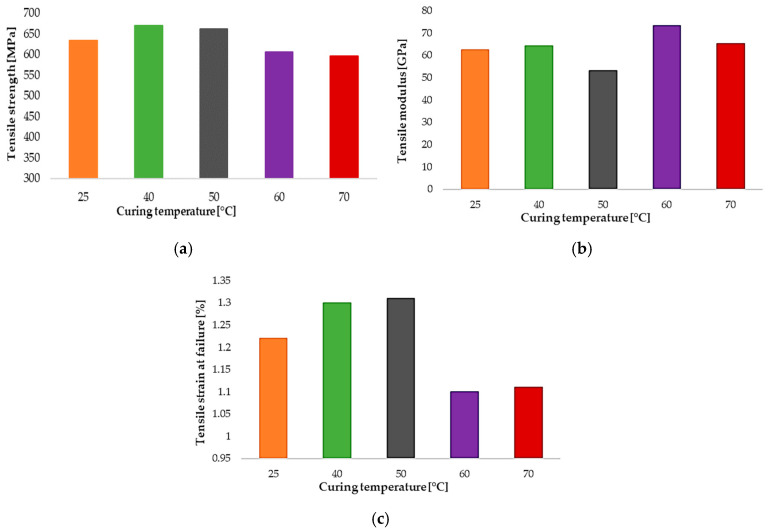
Effect of curing temperature on tensile properties of laminates: (**a**) tensile strength, (**b**) tensile modulus, and (**c**) tensile strain at failure.

At curing temperatures of 60 °C and 70 °C, a reduction in tensile strength is observed despite locally increased elastic modulus values, as shown in Figure 8a,b. This behavior indicates a transition toward a stiffer but more brittle response, as corroborated by the reduced strain at failure reported in Table 1.

The results suggest that excessive matrix crosslinking limits strain accommodation at the fiber–matrix interface, leading to premature damage initiation. Importantly, the tensile strain at failure plotted in Figure 8c reveals a clear reduction for laminates cured at 60 °C and 70 °C. This decrease in strain-to-failure indicates a transition toward a more brittle response at elevated curing temperatures and is consistent with matrix embrittlement and a reduced capacity to accommodate local strain incompatibilities.

### 3.2. Flexural Test

The present work represents a substantial extension and further in-depth investigation of the results previously reported in the study by Stroe et al. (2025) [41], in which only composite laminates cured at the reference temperature of 25 °C and at 50 °C were analyzed. The flexural behavior of the crosslinked composite laminates was assessed by means of three-point flexural tests. For each curing condition, four specimens were tested, and their individual force–displacement and corresponding flexural stress–strain responses were recorded. All laminates exhibited an initial linear elastic regime followed by damage initiation and a post-peak load-bearing response, which is typical for CFRP laminates subjected to flexural loads. The dispersion observed among the curves can be attributed to local variations in laminate thickness, fiber alignment, and damage initiation mechanisms.

**Figure 9:**
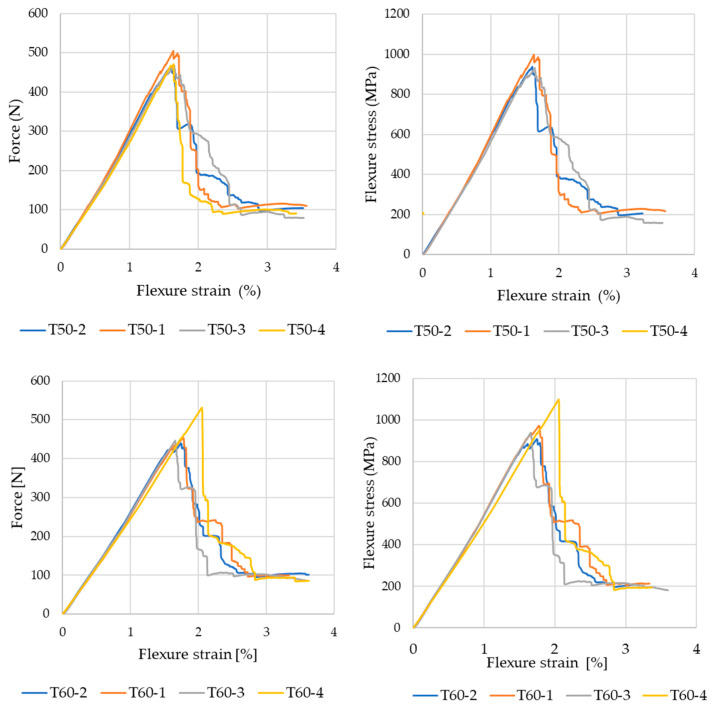
Flexural response of the sample cured at 50 °C and 70 °C: force–strain and flexural stress–strain diagrams.

Representative force–displacement and flexural stress–strain responses for a selected curing condition are shown in Figure 9. To enable a consistent comparison between curing regimes, the flexural properties discussed herein were derived from the average values obtained from the four specimens tested for each laminate. These averaged results were used to determine flexural stiffness, first-peak flexural stress, absorbed energy, and strain at the onset of damage.

**Figure 10:**
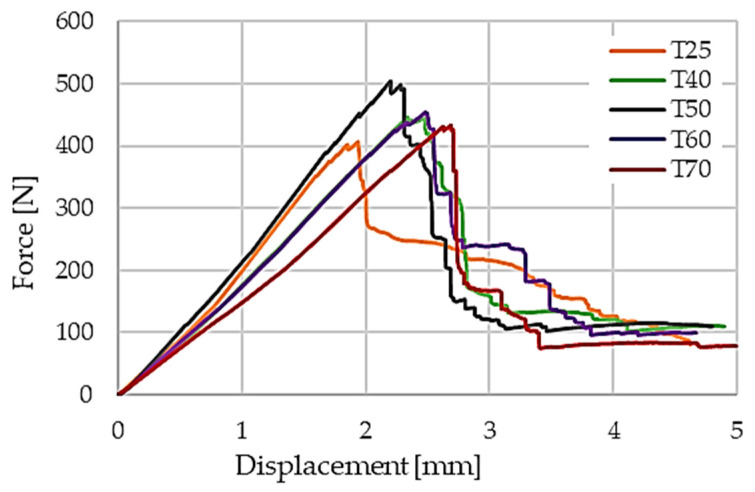
Representative force–displacement curves obtained from three-point bending tests.

**Figure 11:**
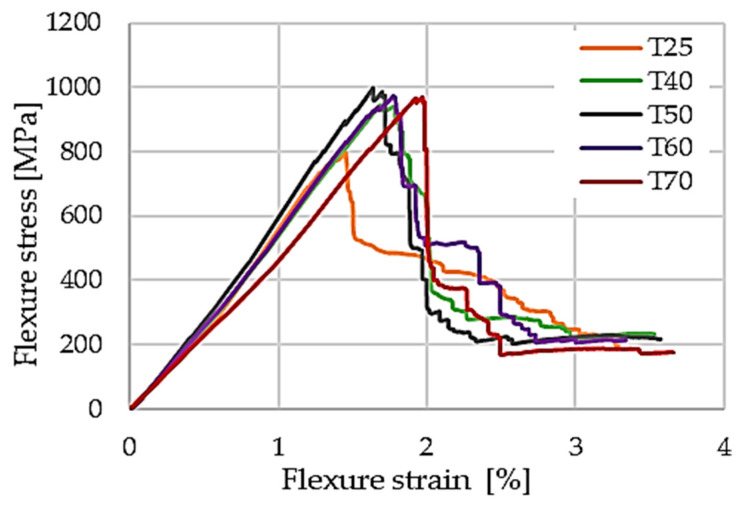
Representative flexural stress–strain curves from three-point bending tests.

The influence of curing temperature on flexural performance is illustrated by the representative force–displacement curves in Figure 10. Laminates cured at 25 °C exhibit reduced initial stiffness and earlier damage initiation, whereas those cured at 40 °C and 50 °C show increased stiffness and delayed damage onset. This trend is further reflected in the flexural stress–strain responses presented in Figure 11, where the laminate cured at 50 °C displays the steepest initial slope and the highest stress at the first damage peak.

**Figure 12:**
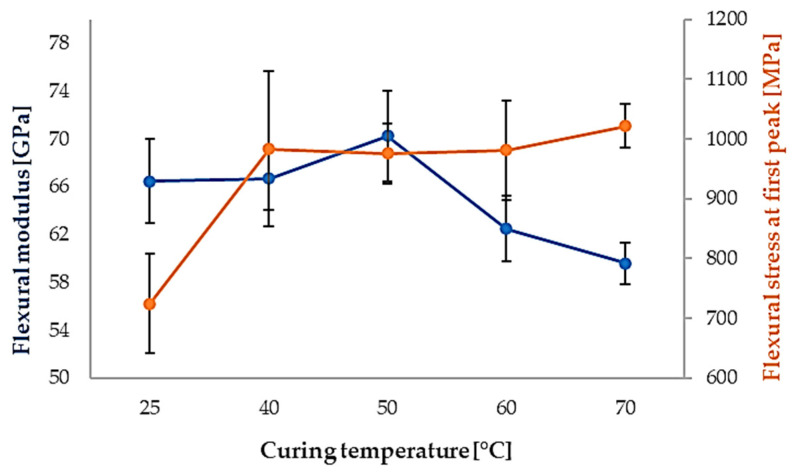
Flexural modulus (blue curve) and flexural stress at first peak (10% of maximum force) (orange curve) as a function of curing temperature.

Quantitative flexural stiffness parameters, including Young’s modulus and secant modulus, are summarized in Table 2, while the corresponding flexural stress, absorbed energy, and strain at the first peak are reported in Table 3. The evolution of flexural modulus and first-peak flexural stress with curing temperature is further highlighted in Figure 12, indicating optimal flexural performance for curing temperatures in the 40–50 °C range.

At higher curing temperatures (60 °C and 70 °C), a decrease in flexural modulus accompanied by increased data scatter is observed, suggesting reduced structural stability and increased sensitivity to local defects.

**Figure 13:**
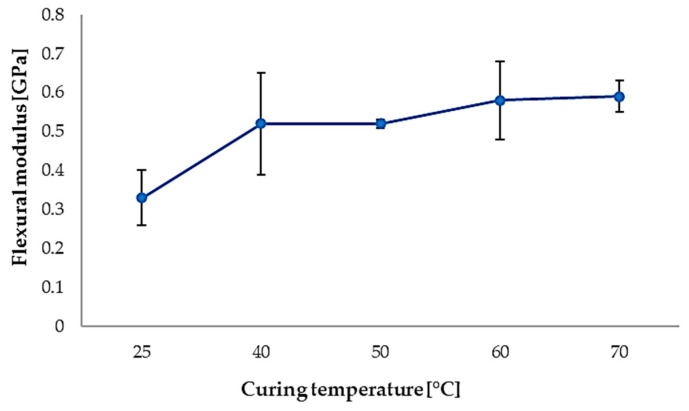
Absorbed energy at the first damage peak as a function of curing temperature.

The absorbed energy at the first damage peak is plotted as a function of curing temperature in Figure 13. A pronounced increase in absorbed energy is observed when moving from 25 °C to 40–50 °C, indicating enhanced resistance to damage initiation and improved stress redistribution within the laminate. Although further increases in absorbed energy are measured for curing temperatures of 60 °C and 70 °C, this trend is accompanied by increased deformation and variability. This behavior suggests that the additional energy absorption at higher curing temperatures is associated with delayed but more unstable damage evolution rather than improved intrinsic toughness.

### 3.3. Heat Deflection Temperature (HDT) Test

**Figure 14:**
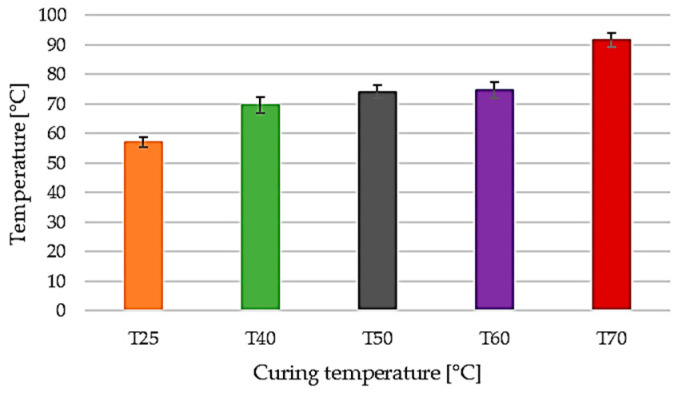
Thermo-mechanical response of samples during HDT testing: heat deflection temperature as a function of curing temperature.

The thermo-mechanical behavior of the laminates was assessed through heat deflection temperature (HDT) measurements. The evolution of HDT as a function of curing temperature is presented in Figure 14, while the corresponding numerical values are summarized in Table 4. A clear monotonic increase in HDT is observed with increasing curing temperature, indicating progressive stiffening of the epoxy matrix and enhanced resistance to thermal softening under mechanical load.

The laminate cured at room temperature (25 °C) exhibits the lowest HDT value, 57.01 °C, reflecting early softening of the polymer matrix. In contrast, thermally assisted curing leads to a significant improvement in thermo-mechanical stability, with HDT values increasing to 70.25 °C for the 40 °C curing condition and reaching approximately 74 °C for laminates cured at 50 °C and 60 °C. These results confirm the strong influence of curing temperature on the thermo-mechanical performance of the composite laminates.

The most significant enhancement is recorded for the laminate cured at 70 °C, which reaches an HDT of 92.15 °C. This substantial increase reflects the development of a denser and more rigid epoxy network, capable of sustaining the applied load up to considerably higher temperatures before the onset of thermal deflection. However, when the HDT results are interpreted alongside the mechanical data reported in Table 4 and Figure 14, it becomes evident that higher thermal stability does not necessarily translate into superior mechanical performance under quasi-static loading conditions.

### 3.4. Differential Scanning Calorimetry (DSC) Analyses

Differential scanning calorimetry (DSC) was employed to investigate the thermal behavior and network development of the epoxy matrix as a function of curing temperature. Measurements were performed on cured L20 epoxy resin extracted from the composite laminates, using a cooling–heating protocol in the temperature range of 30–200 °C. This approach enables the separation of reversible glass transition phenomena from irreversible thermal history effects, such as physical aging and enthalpy relaxation [42,43].

**Figure 15:**
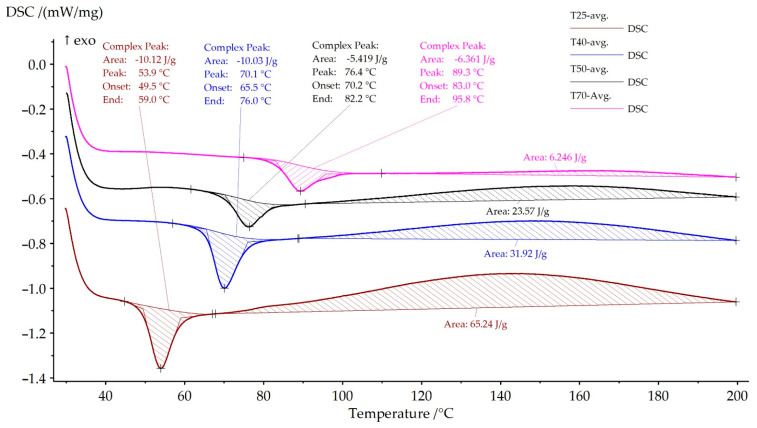
DSC heating curves of the cured epoxy resin showing the glass transition region and the associated enthalpy relaxation behavior as a function of curing temperature.

The heating curves primarily reflect the prior thermal history of the material and provide insight into the extent of physical aging and incomplete structural relaxation. As shown in Figure 15, all samples exhibit a distinct endothermic feature within the glass transition region, attributed to enthalpy relaxation. This phenomenon arises from the release of stored internal energy accumulated during storage below Tg and is commonly observed in partially relaxed epoxy networks [43,44].

With increasing curing temperature, the enthalpy relaxation peak progressively decreases in magnitude and shifts toward higher temperatures (Figure 15). The specimen cured at 25 °C shows the most pronounced relaxation peak, indicating a higher degree of residual segmental mobility and incomplete network relaxation. In contrast, the sample cured at 70 °C exhibits only a weak relaxation feature, consistent with a polymer network closer to thermodynamic equilibrium and a higher degree of structural stabilization.

A clear correlation is observed between the enthalpy relaxation temperature extracted from the DSC heating curves (Figure 15) and the experimentally measured heat deflection temperature (HDT). For the sample cured at 25 °C, the relaxation peak occurs at approximately 53.9 °C, which closely matches the corresponding HDT value of 57.01 ± 1.57 °C. As the curing temperature increases to 40 °C and 50 °C, the relaxation temperature shifts to approximately 70.1 °C and 76.4 °C, respectively, accompanied by a systematic increase in HDT. The highest curing temperature investigated (70 °C) results in the highest relaxation temperature (~89.3 °C) and the maximum HDT value (92.15 ± 2.45 °C). Since both enthalpy relaxation and HDT are governed by the onset of cooperative segmental mobility in the glass transition region, this correlation is expected for epoxy systems [43,45]. A quantitative summary of these relationships is provided in Table 5.

**Figure 16:**
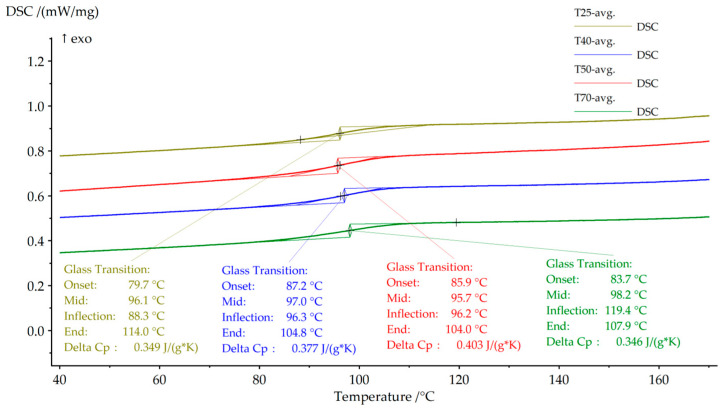
DSC cooling curves of cured L20 epoxy resin for different curing temperatures, illustrating the glass transition temperature (Tg) and the associated heat capacity change (ΔCp).

To obtain glass transition temperatures that are independent of enthalpy relaxation and prior thermal history effects, Tg values discussed in this work are derived exclusively from the DSC cooling curves. The cooling responses corresponding to the different curing conditions are presented in Figure 16, where Tg is determined as the midpoint of the heat capacity change.

As illustrated in Figure 16, Tg increases modestly from approximately 96.1 °C for the room-temperature cured sample to about 98.2 °C for the specimen cured at 70 °C. Although the absolute variations are relatively small, the overall trend indicates a gradual stiffening and homogenization of the epoxy network with increasing curing temperature. Minor deviations, such as the slightly lower Tg observed for the 50 °C curing condition, can be attributed to local variations in crosslink distribution, which are typical for thermoset systems cured under intermediate thermal conditions.

Additional insight into the evolution of the epoxy network is provided by the heat capacity increment at the glass transition (ΔCp), also extracted from the cooling curves (Figure 16). As summarized in Table 6, ΔCp increases from approximately 0.35 J·g^−1^·K^−1^ for the room-temperature cured sample to values approaching 0.38–0.40 J·g^−1^·K^−1^ for thermally assisted curing conditions. An increased ΔCp is commonly associated with reduced structural heterogeneity and a more uniformly developed polymer network, indicating that curing on heated surfaces promotes improved network formation [43,45].

Overall, the combined analysis of DSC heating and cooling curves demonstrates that increasing the curing (soaking) temperature is more effective than extending curing time at low temperatures for promoting network stabilization, reducing physical aging effects, and enhancing thermo-mechanical performance [42,46]. Compared to prolonged curing at ambient temperature, thermally assisted curing on temperature-controlled heated surfaces enables superior network development within shorter processing times.

From a sustainability perspective, the curing strategy based on temperature-controlled heated surfaces offers clear advantages compared to prolonged curing at ambient temperature. The experimental results demonstrate that curing on heated surfaces at moderate temperatures (40–70 °C) for reduced holding times (12 h) leads to systematic improvements in both thermal and thermo-mechanical performance, as evidenced by the increase in glass transition temperature (Tg) derived from DSC cooling curves and the significant rise in heat deflection temperature (HDT).

The use of heated surfaces enables localized and targeted heat input, improving thermal efficiency by limiting unnecessary heat dissipation to the surrounding environment. Compared to extended curing at room temperature (24 h), thermally assisted curing on heated surfaces achieves superior network development within a shorter processing time, thereby reducing overall energy demand while maintaining or enhancing material performance. The concurrent increase in Tg, reduction in enthalpy relaxation intensity, and upward shift in the enthalpy relaxation temperature indicate a more stable and better-developed epoxy network when curing temperature is appropriately increased.

When optimized within the investigated temperature range, this curing approach represents a viable and more sustainable processing route for epoxy-based composite systems, enabling improved thermo-mechanical performance without the need for high-temperature oven curing or extended curing durations.

## 4. Conclusions

This study investigated the influence of curing temperature on the mechanical, thermo-mechanical, and thermal behavior of out-of-autoclave (OoA) carbon-fiber-reinforced epoxy laminates manufactured by resin infusion and post-cured on heated surfaces. Tensile and flexural testing, combined with HDT measurements and DSC analyses, enabled a clear correlation between thermal transitions and mechanical performance.

The results show that curing temperature critically governs both laminate-level properties and matrix-level thermal behavior. Laminates cured at moderate temperatures (40–50 °C) exhibit the best balance between tensile/flexural performance and thermal stability, while higher curing temperatures (60–70 °C) increase thermal stability (HDT up to 92 °C) but reduce ductility and tensile strength, indicating embrittlement due to excessive cross-linking. DSC cooling curves demonstrate a systematic increase in Tg with curing temperature (up to 98.2 °C at 70 °C), while DSC heating curves reveal enthalpy relaxation shifts consistent with reduced segmental mobility and more homogeneous network formation.

Importantly, a direct correlation is established between enthalpy relaxation temperature, Tg, HDT, and mechanical behavior, confirming that optimal curing conditions promote sufficient crosslink density and network homogeneity for improved structural performance, whereas Tg alone is not a sufficient predictor of mechanical properties. The results identify 40–50 °C as the optimal post-curing window for the L20/H161 system, where mechanical strength, flexural response, and thermal stability are simultaneously maximized.

Additionally, the OoA curing approach demonstrates sustainability advantages compared to prolonged ambient or high-temperature oven/autoclave curing. The use of moderate post-curing on heated surfaces reduces energy consumption, shortens processing time, and limits heat losses, supporting a more energy-efficient and environmentally friendly manufacturing route without compromising composite performance.

Overall, this integrated approach highlights the necessity of combining mechanical, thermo-mechanical, and thermal analyses when defining curing strategies for OoA composites. Future work will extend DSC analyses and systematically explore the combined effect of curing temperature and soaking time to further optimize crosslink density, laminate performance, and processing efficiency.

## Figures and Tables

**Table 1:** Tensile properties of laminates cured at different temperatures.

Curing Temperature [°C]	Tensile Strength [MPa]	Tensile Modulus [GPa]	Tensile Strain at Failure [%]
25	632.6 ± 23.7	62.4 ± 8.9	1.22 ± 0.08
40	668.7 ± 41.6	64.2 ± 7.5	1.30 ± 0.09
50	661.5 ± 11.6	53.2 ± 1.9	1.31 ± 0.05
60	604.8 ± 46.7	73.2 ± 13.0	1.10 ± 0.08
70	595.7 ± 28.6	65.2 ± 9.8	1.11 ± 0.11

**Table 2:** Flexural stiffness parameters of laminates cured at different temperatures.

Curing Temperature [°C]	Flexural Modulus (Young’s) [GPa]	Secant Modulus (0.5–1% Strain) [GPa]
25	66.46 ± 3.55	62.78 ± 2.77
40	66.68 ± 2.67	61.42 ± 1.94
50	70.25 ± 3.80	62.70 ± 2.50
60	62.46 ± 2.75	55.58 ± 2.66
70	59.59 ± 1.75	51.77 ± 4.04

**Table 3:** Flexural performance at the first peak (10% of maximum force).

Curing Temperature [°C]	Flexural Stress at First Peak [MPa]	First Peak Force [N]	Energy at First Peak [J]	Flexural Strain at First Peak [%]
25	724.50 ± 83.87	363.31 ± 42.78	0.33 ± 0.07	1.30 ± 0.14
40	983.14 ± 130.51	435.20 ± 56.38	0.52 ± 0.13	1.67 ± 0.20
50	975.52 ± 49.89	477.31 ± 18.26	0.52 ± 0.01	1.64 ± 0.02
60	980.61 ± 83.74	467.81 ± 42.79	0.58 ± 0.10	1.81 ± 0.17
70	1021.69 ± 37.15	447.94 ± 11.64	0.59 ± 0.04	1.92 ± 0.06

**Table 4:** Heat deflection temperature of samples cured at different temperatures.

Curing Temperature [°C]	Applied Load [MPa]	HDT [°C]
25	0.45	57.01 ± 1.57
40	0.45	70.25 ± 2.65
50	0.45	73.65 ± 2.25
60	0.45	75.25 ± 2.75
70	0.45	92.15 ± 2.45

**Table 5:** Correlation between DSC-derived parameters and heat deflection temperature (HDT).

Curing Temperature (°C)	Enthalpy Relaxation Temperature (°C)	HDT (°C)
25	~53.9	57.01 ± 1.57
40	~70.1	70.25 ± 2.65
50	~76.4	73.65 ± 2.25
70	~89.3	92.15 ± 2.45

**Table 6:** DSC parameters obtained from cooling curves for cured L20 epoxy resin.

Curing Temperature (°C)	Curing Time (h)	Tg (Cooling, °C)	ΔCp at Tg (J·g^−1^·K^−1^)
25	24	96.1	~0.35
40	12	97	~0.38
50	12	95.7	~0.40
70	12	98.2	~0.35

## Data Availability

The original contributions presented in the study are included in the article, further inquiries can be directed to the corresponding author.

## Abbreviations

The following abbreviations are used in this manuscript:

OoA	Out-of-Autoclave
CFRP	Carbon-fiber-reinforced polymers
DSC	Differential scanning calorimetry
HDT	Heat deflection temperature
Tg	Glass transition temperature
ΔCp	Heat capacity change
